# Priming of Adult Incision Response by Early-Life Injury: Neonatal Microglial Inhibition Has Persistent But Sexually Dimorphic Effects in Adult Rats

**DOI:** 10.1523/JNEUROSCI.1786-18.2019

**Published:** 2019-04-17

**Authors:** Orla Moriarty, YuShan Tu, Ameet S. Sengar, Michael W. Salter, Simon Beggs, Suellen M. Walker

**Affiliations:** ^1^Developmental Neurosciences Programme (Pain Research), University College London Great Ormond Street Institute of Child Health, London WC1N 1EH, United Kingdom,; ^2^Neurosciences and Mental Health Program, Hospital for Sick Children, Department of Physiology, Faculty of Medicine, University of Toronto, Toronto, Ontario M5G 1X8, Canada,; ^3^Neuroscience Physiology and Pharmacology, University College London, London WC1E 6BT, United Kingdom, and; ^4^Department of Anaesthesia and Pain Medicine, Great Ormond Street Hospital, National Health Service Foundation Trust, London WC1N 3JH, United Kingdom

**Keywords:** dorsal horn, incision, microglia, neonate, pain, sex-dependent

## Abstract

Neonatal hindpaw incision primes developing spinal nociceptive circuitry, resulting in enhanced hyperalgesia following reinjury in adulthood. Spinal microglia contribute to this persistent effect, and microglial inhibition at the time of adult reincision blocks the enhanced hyperalgesia. Here, we pharmacologically inhibited microglial function with systemic minocycline or intrathecal SB203580 at the time of neonatal incision and evaluated sex-dependent differences following adult reincision. Incision in adult male and female rats induced equivalent hyperalgesia and spinal dorsal horn expression of genes associated with microglial proliferation (*Emr1*) and transformation to a reactive phenotype (*Irf8*). In control adults with prior neonatal incision, the enhanced degree and duration of incision-induced hyperalgesia and spinal microglial responses to reincision were equivalent in males and females. However, microglial inhibition at the time of the neonatal incision revealed sex-dependent effects: the persistent mechanical and thermal hyperalgesia following reincision in adulthood was prevented in males but unaffected in females. Similarly, reincision induced *Emr1* and *Irf8* gene expression was downregulated in males, but not in females, following neonatal incision with minocycline. To evaluate the distribution of reincision hyperalgesia, prior neonatal incision was performed at different body sites. Hyperalgesia was maximal when the same paw was reincised, and was increased following prior incision at ipsilateral, but not contralateral, sites, supporting a segmentally restricted spinal mechanism. These data highlight the contribution of spinal microglial mechanisms to persistent effects of early-life injury in males, and sex-dependent differences in the ability of microglial inhibition to prevent the transition to a persistent pain state span developmental stages.

**SIGNIFICANCE STATEMENT** Following the same surgery, some patients develop persistent pain. Contributory mechanisms are not fully understood, but early-life experience and sex/gender may influence the transition to chronic pain. Surgery and painful procedural interventions in vulnerable preterm neonates are associated with long-term alterations in somatosensory function and pain that differ in males and females. Surgical injury in neonatal rodents primes the developing nociceptive system and enhances reinjury response in adulthood. Neuroimmune interactions are critical mediators of persistent pain, but sex-dependent differences in spinal neuroglial signaling influence the efficacy of microglial inhibitors following adult injury. Neonatal microglial inhibition has beneficial long-term effects on reinjury response in adult males only, emphasizing the importance of evaluating sex-dependent differences at all ages in preclinical studies.

## Introduction

Early-life stress, adversity, and pain can influence neurodevelopmental and health outcomes throughout the lifespan (Nemeroff, 2016; Burke et al., 2017). The need to understand how early-life experience influences chronic pain in later life has been highlighted (Price et al., 2018). In preterm-born neonates, surgery and repeated painful procedures during intensive care are associated with worse neurodevelopmental outcome (Ranger and Grunau, 2014; Hunt et al., 2018), altered brain structure (Duerden et al., 2018), and differences in somatosensory function and pain experience during childhood (Hermann et al., 2006; Walker et al., 2009b) that persist, but with sex differences emerging in young adults (Walker et al., 2018). While analgesia improves acute outcome, the long-term benefit of neonatal analgesic interventions is debated (Walker, 2017; Schiller et al., 2018). Evaluating mechanisms triggered by early-life tissue injury is essential to identify preventive strategies that minimize long-term alterations in pain response.

Microglia are critical mediators of normal development, sculpting neuronal circuitry in the developing CNS, and being implicated in diverse functions, including neurogenesis, synaptic pruning, and synaptic plasticity (Kettenmann et al., 2013; Salter and Stevens, 2017). Early-life stress and tissue injury can disrupt microglial sex-dependent maturation or trigger long-term changes in microglial phenotype, altering reactivity to future immune or environmental challenges and influencing responses to physical and psychological stressors and susceptibility to neurological disorders (Perry and Holmes, 2014; Burke et al., 2016; Hanamsagar and Bilbo, 2017). While brain injury secondary to hypoxia/ischemia, hyperoxia, or trauma in neonatal rodents evokes a neuroinflammatory response and increased microglial reactivity, the pathophysiological role of microglia can vary with type of injury, time, and brain region (Hagberg et al., 2015; Salter and Stevens, 2017). Microglial inhibition with minocycline has also been variably reported to have no benefit (Cikla et al., 2016), paradoxically increase acute cell death (Strahan et al., 2017) and worsen long-term function (Hanlon et al., 2017), or improve outcome (Wixey et al., 2011; Schmitz et al., 2014), depending on the assessment method, type, and age of brain injury.

Plantar hindpaw incision during the first postnatal week in the rat produces activity-dependent alterations in adult sensory threshold and increased hyperalgesia when the paw is reincised (Walker et al., 2009a; Moriarty et al., 2018). As this enhanced reincision hyperalgesia is reduced by microglial inhibitors (intrathecal minocycline or p38 inhibitor) in adult males (Beggs et al., 2012; Schwaller et al., 2015), we hypothesized that spinal microglia are involved in priming spinal nociceptive signaling and amplifying the subsequent injury response. Neuroimmune signaling is sexually dimorphic (Gutierrez et al., 2013; Nelson et al., 2018), and microglial inhibitors in male, but not female, adult rodents reduce pain behaviors following peripheral nerve injury, hindpaw inflammation (Sorge et al., 2011, 2015; Taves et al., 2016; Mapplebeck et al., 2018), and hyperalgesic priming (Paige et al., 2018). As the efficacy of mechanism-based interventions may differ in males and females, and sex-dependent differences in experimental pain sensitivity and chronic pain prevalence are well documented in clinical populations (Mogil, 2012; Fillingim, 2017), considering sex as a biological variable (Shansky and Woolley, 2016) is particularly relevant for preclinical pain studies (Rosen et al., 2017). In addition, evaluation following neonatal tissue injury is required to identify developmentally regulated and persistent effects of early-life pain.

To further investigate the contribution of spinal microglia to persistent and potentially sex-dependent differences in pain response following early-life injury, we now adopted a preventive strategy. Microglial inhibitors (systemic minocycline or intrathecal p38 inhibitor SB203580) were administered concurrently with neonatal plantar hindpaw incision, and our primary outcome was the impact on reincision hyperalgesia in adult male and female rats. To assess incision-induced spinal microglial response, expression of genes related to microglial reactivity and proliferation were assessed in adult males and females. Finally, to determine whether reincision hyperalgesia is restricted to the prior incision site or has a segmental distribution, neonatal incision was performed at different body sites, and hyperalgesia was compared following left hindpaw incision in adulthood.

## Materials and Methods

### 

#### 

##### Animals.

All procedures were performed under personal and project licenses approved by the United Kingdom Home Office in accordance with the Animal (Scientific Procedures) Act, 1986 or with the approval of the Animal Care Committee of the Hospital for Sick Children, Toronto and in accordance with the Canadian Council on Animal Care. Reporting of results follows the ARRIVE Guidelines (Kilkenny et al., 2010). Behavioral and electrophysiology experiments were performed in the United Kingdom with Sprague Dawley (RRID:MGI:5651135) adult rats and litters of rat pups obtained from the same colony, bred and maintained in-house by the Biological Services Unit, University College London. Handling of rat pups and duration of maternal separation were kept to a minimum, with body temperature maintained on a heating blanket. Spinal cord gene expression studies were performed in Toronto, with additional Sprague Dawley rats obtained from Charles River Laboratories. All animals were regularly monitored and maintained under standard environmental conditions with food and water available *ad libitum*. All procedures were performed during the light phase (12 h light/dark cycle, lights on 08:00–20:00 h). Individual litters were reduced to a maximum of 12 pups and weaned into same-sex cages at postnatal day (P) 21. Experimental groups comprised male and female rats distributed across multiple litters and/or adult cage groups (4 or 5/cage). Each rat was considered an experimental unit, except in the case of PCR tissue analysis where 2 animals were pooled per unit. Rats were randomly selected from the litter or cage, numbered, and then allocated to treatment groups according to a computer-generated randomization code. Animals and tissue samples were coded by an independent colleague to ensure the experimenter was unaware of treatment allocation during behavioral testing or tissue analysis.

##### Surgical procedures.

All procedures were performed under isoflurane (Isoflo, Abbott) anesthesia (2%–4% in 1 L/min oxygen). Plantar hindpaw incision was chosen as a clinically relevant and established model of surgical injury in infant and adult rodents (Brennan et al., 1996) with incision of skin and muscle producing acute hyperalgesia and increased spinal excitability at all ages (Ririe et al., 2003, 2008), and activity-dependent long-term alterations in spinal reflex sensitivity, synaptic signaling, and response to reinjury (Walker et al., 2009a; Li et al., 2015; Li and Baccei, 2016). Skin incisions in neonatal (P3) and adult (6–8 weeks age) rats were matched to the relative length of the hindpaw from the midpoint of the heel to the first skin pad as previously described (Beggs et al., 2012), with elevation and longitudinal incision of underlying plantaris muscle using a number 11 blade scalpel. Neonatal nonincision minocycline or saline controls had injections performed with the same depth and duration of anesthesia as incision groups, and the same degree of handling and duration of maternal separation. We have previously shown that the response to adult incision does not differ between littermates with prior neonatal handling and anesthesia and naive age-matched adults (Beggs et al., 2012).

Neonatal incisions were also performed at different sites: ipsilateral (left) hindpaw; contralateral (right) hindpaw; and the left and right forepaw. Forepaw and hindpaw sizes are more comparable in pups than adult rats, and we have previously shown that incision at either site produces the same degree of acute hyperalgesia (Walker et al., 2015). As microglial reactivity in the lateral dorsal horn was increased following thigh incision for exposure of the sciatic nerve in adult rats with prior neonatal incision (Beggs et al., 2012), we also performed neonatal incisions on the left anterior thigh based on the skin-muscle incision and retraction model (Flatters, 2008), but without retraction to minimize tissue damage in neonatal animals.

All adult incisions were performed on the plantar surface of the left hindpaw. Incisions were closed in rat pups with a single loop of 5-0 silk suture (Mersilk #W595, Ethicon) to produce small stable knots in pups, and with two mattress 5-0 silk sutures in adult animals to standardize the material at both ages. Animals were monitored daily to ensure skin closure remained intact and residual sutures were removed at 5 d. Pups were maintained on a warming blanket and returned to the dam following recovery from anesthesia or between evaluations.

##### Drug administration.

All injections of drug or control solutions were performed under brief isoflurane (Isoflo) anesthesia (2%–4% in 1 L/min oxygen). Minocycline hydrochloride (Sigma-Aldrich, catalog #M9511) was diluted to 4 mg/ml in sterile saline and administered by intraperitoneal injection. P3 rats received 45 mg/kg minocycline 30 min before incision, and 22.5 mg/kg on day 1 (P4) and 2 (P5) after incision, as neonatal rats have previously been shown to tolerate this dose regimen (Buller et al., 2009; Wixey et al., 2011). Control animals received an equivalent volume of saline.

The p38 mitogen-activated protein kinase (MAPK) inhibitor 4-(4-fluorophenyl)-2-(4-methylsulfonylphenyl)-5-(4-pyridyl)-1H-imidazole(SB203580; EMD Millipore, catalog #19-135) was solubilized in DMSO (Sigma-Aldrich D2650 Hybri-Max sterile-filtered, PubChem ID 24893703) and then diluted to a final concentration of 0.8 mg/ml in 8% DMSO. We have previously shown that intrathecal SB203850 1 mg/kg reduces mechanical hyperalgesia and spinal microglial expression of phosphorylated-p38 following hindpaw incision in adult rats (Schwaller et al., 2015). Here, percutaneous low lumbar injections were performed by the same investigator (S.M.W.) as previously described (Walker et al., 2010), with divided doses to match the timing of minocycline experiments: 0.4 mg/kg SB203850 (injectate volume 0.5 μl/g) 30 min before P3 incision, and 0.3 mg/kg SB203850 on day 1 and 2 after incision. As the developing spinal cord is susceptible to high local concentrations of some drugs and diluents (Walker and Yaksh, 2012), vehicle control animals received an equivalent volume of 8% DMSO.

##### Behavioral testing.

In rat pups, hand-held calibrated von Frey filaments (0.13–7.8 g) were sequentially applied five times at 1 s intervals and the number of evoked flexion reflexes recorded. The maximum force applied was that which evoked five withdrawal responses. A sigmoidal stimulus–response curve was generated for each animal with the midpoint (50% effective force [EF_50_]) calculated as the threshold (Walker et al., 2009a).

Adult rats were habituated on an elevated mesh platform for 1 h before testing. An electronic von Frey device (Dynamic Plantar Aesthesiometer, catalog #37450) applied increasing pressure to the plantar hindpaw (20 g/s to a maximum of 50 g), and mechanical withdrawal threshold was calculated as the mean of three measures of the force producing brisk hindlimb withdrawal. For thermal latency, animals were habituated to the heated glass surface of a modified Hargreaves apparatus (University Anesthesia Research and Development Group, University of California San Diego, La Jolla, CA), and the time for withdrawal from a heat stimulus directed at the mid-plantar paw was recorded (maximum 20 s). The mean of three measures was designated as thermal withdrawal latency.

For evaluation of spontaneous locomotor activity, animals were habituated to an open field consisting of a 90 cm square dark gray plastic arena (40 cm high) for 20 min on the day before adult incision, and testing was performed 48 h later (24 h after incision). A video camera placed above the open field tracked movement over a 3 min period for subsequent analysis with Ethovision behavioral tracking software (version XT 11, Noldus, RRID:SCR_004074).

##### Electromyography (EMG) recording.

Flexor reflex EMG recordings were performed 24 h after incision in neonatal and adult rats (Walker et al., 2009a). Briefly, animals were anesthetized (2%–4% isoflurane in 1 L/min oxygen), and the trachea cannulated for mechanical ventilation (Small Animal Ventilator, Harvard Apparatus). The inspired isoflurane concentration was reduced to 1.75% in P4 pups and 1.25% in adult rats for 20 min to allow equilibration and was maintained at this level to provide stable anesthesia during EMG recordings. The left hindpaw was secured with an adhesive pad on a fixed platform and a bipolar EMG electrode comprising a stainless-steel 30 gauge needle with a central copper wire core was placed through a small skin incision into the biceps femoris muscle. von Frey hairs were applied to the plantar surface of the hindpaw for 1 s, and the EMG response to the mechanical stimulus was processed (Neurolog, Digitimer) and recorded in 12 s epochs (PowerLab 4S, AD Instruments, RRID:SCR_001620). To evaluate responses to both threshold and suprathreshold stimuli, von Frey hairs were sequentially applied up to a maximum 60 g bending force (von Frey hair number 17) at P4, and 180 g (von Frey hair number 20) in adults, with a minimum of 60 s between stimuli. The duration of the EMG response was outlined from the display of the raw data, and the integral of the root mean square of the signal was calculated (EMG response, Chart, PowerLab, AD Instruments). The EMG response was plotted against the von Frey hair number (mechanical stimulus) and the area under the stimulus–response curve calculated to quantify the overall “reflex response” (Walker et al., 2009a).

##### Tissue preparation and analysis.

Rats were terminally anesthetized with pentobarbital (100 mg/kg i.p., Euthatal, Merial Animal Health) and transcardially perfused with heparinized saline followed by 4% PFA (Thermo Fisher Scientific, catalog #10131580). Spinal cords were exposed, and the L4 to L5 spinal segment dissected. Tissue was postfixed in 4% PFA, then cryoprotected in sucrose (30% sucrose, 0.02% sodium azide in 0.1 m PB).

Neonatal L4/L5 spinal 20 μm free-floating sections were mounted on SuperFrost slides (Thermo Fisher Scientific, catalog #10149870). P4 cords (24 h after intervention) were assessed for cell death with Fluoro-Jade C (FJ-C) staining, and P6 cords (3 d after intervention) for microglial cell counts with ionized calcium binding adaptor molecule (Iba1) immunohistochemistry.

For Iba1 immunohistochemistry, sections were washed initially and between subsequent steps with PBS containing 0.1% Triton X-100, blocked for 1 h at room temperature (5% chicken serum in PBS), and then incubated for 24 h with primary goat anti-Iba1 antibody (1:400, Abcam, catalog #ab5076, RRID:AB_2224402) followed by AlexaFluor-594-conjugated chicken anti-goat IgG (1:200, Invitrogen, catalog #A-21468, RRID:AB_2535871) for 24 h at room temperature. Sections were coverslipped with Prolong Gold fluorescent mounting media (Invitrogen, catalog #P36930, RRID:SCR_015961). Negative controls omitting the primary antibody resulted in a complete absence of positive staining.

For FJ-C staining, slides were stained in the following sequence: washed in 0.1 m PB, immersed in 1% sodium hydroxide in 80% ethanol, rinsed with 70% ethanol then distilled water, incubated in 0.06% potassium permanganate for 10 min, stained with 0.0002% FJ-C (Millipore, catalog #AG325-30MG) and 0.0001% DAPI (Invitrogen, catalog #D1306, RRID:AB_2629482) prepared in 0.1% acetic acid, and then cleared and coverslipped.

Sections were visualized at 10× magnification for fluorescence (DMR, Leica Microsystems) under FITC or TRITC filters, and images obtained using a Hamamatsu (ORCA-100 C4742-95) digital camera. Iba1-positive cells within a standard size region of interest over the medial dorsal horn were counted (ImageJ software, https://imagej.nih.gov/ij/, RRID:SCR_003073, cell-counter plugin). FJ-C-positive cells were counted in each quadrant (dorsal: ipsilateral and contralateral; ventral: ipsilateral and contralateral). All counts were averaged for a minimum of 6 sections (Iba1), or summed for 6 sections (FJ-C), per rat, and *n* indicates the number of rats per group.

Three days following adult incision, animals for qPCR experiments were transcardially perfused with ∼30 ml of RNAlater (Ambion, Invitrogen, catalog #AM7021), and a cylindrical biopsy tissue punch (2.0 mm internal diameter, Harvard Apparatus, catalog #72-5041) was used to isolate tissue from the ipsilateral L5 medial dorsal horn that was stored in RNAlater until processing.

For qRT-PCR, tissue from 2 animals was pooled for each experimental unit, and total RNA was extracted using a PureLink RNA mini kit (Ambion, Invitrogen, catalog #12183018A). The quantity, purity, and quality of RNA were assessed with an ND-2000 Nanodrop spectrophotometer. Samples were equalized to a concentration of 250 ng/20 μl by addition of RNAase free water, and RNA extracts were reverse transcribed to cDNA using the SuperScript VILO cDNA kit (Invitrogen, catalog #11754050). Gene expression of target proteins was determined using commercially available TaqMan gene expression assays (Applied Biosystems, catalog #4331182) containing specific forward and reverse target primers and FAM-labeled MGB probes. Assay IDs for the genes investigated were as follows: *Emr1*, Rn01527631_m1; *Irf8*, Rn01762214_m1. qPCRs were run with 12.5 ng of cDNA and TaqMan Master Mix (Applied Biosystems, catalog #4324018) on a StepOne Plus real-time PCR machine (Invitrogen, catalog #4376600, RRID:SCR_015805) using the following parameters: one cycle of 95°C for 20 s, followed by 40 cycles at 95°C for 1 s and 60°C for 20 s. Reactions were performed in triplicate, and nontemplate controls were included in each run. Amplification plots and copy threshold (C_t_) values were examined using StepOne software (version 2.3, Invitrogen, RRID:SCR_014281). Expression was normalized to the average of three housekeeping genes (*Abt1*, *Eef2*, and *GAPDH*). Relative gene expression was calculated using the ΔΔCt method, and data are expressed relative to the naive or the saline-treated double incision group.

##### Experimental design.

**Figure 1.**
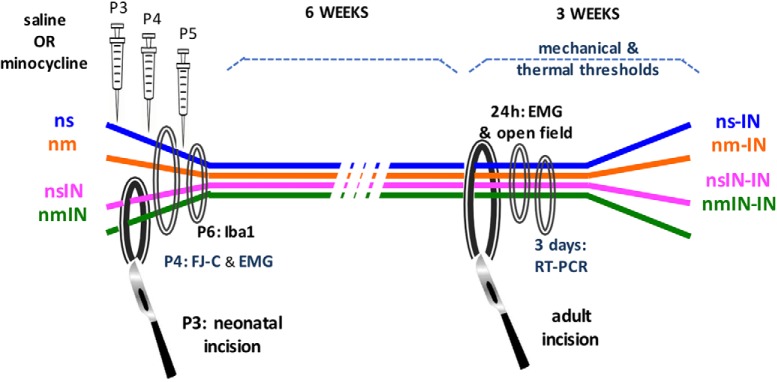
Schematic of experimental design. Treatment groups included the following: ns, nm, nsIN, and nmIN. Injections were performed on P3, P4, and P5. All animals the underwent incision in adulthood (ns-IN, nm-IN, nsIN-IN, nmIN-IN). Evaluations included the following: measures of reflex sensitivity with mechanical withdrawal threshold, thermal withdrawal latency and EMG recordings; spontaneous activity in open field; neonatal spinal tissue analysis with FJ-C staining and Iba1 immunohistochemistry; and spinal gene expression with qRT-PCR following adult incision. In additional experiments, treatment groups included intrathecal injection at the same neonatal time points of 8% DMSO vehicle (nv), and neonatal incision with vehicle (nvIN) or SB203580 (nSBIN). Mechanical withdrawal thresholds were compared following incision 6–7 weeks later (nv-IN vs nSBIN-IN vs nvIN-IN).

To assess the long-term impact of microglial inhibition restricted to the time of neonatal incision, and minimize potential disruption of normal development, pharmacological microglial inhibitors were administered 30 min before incision and at 24 and 48 h. Male and female P3 rat pups were randomly assigned to four experimental groups: neonatal saline (ns), neonatal minocycline (nm), neonatal saline plus incision (nsIN), and neonatal minocycline plus incision (nmIN) (Fig. 1). The number of animals per group was based on our previous studies using similar methodology (Beggs et al., 2012; Schwaller et al., 2015). Several outcomes were compared across treatment groups. (1) Changes in hindlimb reflex withdrawal assessed behavioral hyperalgesia. In neonatal animals, mechanical withdrawal thresholds were measured at baseline on P3, and then 4, 24, 48, and 72 h after intervention. At 6–8 weeks of age, baseline mechanical threshold and thermal withdrawal latency were measured before and at regular intervals to 21 d after left hindpaw incision (ns-IN, nm-IN, nsIN-IN, and nmIN-IN). (2) EMG recordings in anesthetized animals quantified reflex sensitivity 24 h after neonatal or adult incision. (3) Spontaneous locomotor activity was assessed in adults by movement in an open field 24 h following incision. (4) Tissue analysis was performed on lumbar spinal cord. In neonates, sections were collected on P4 for FJ-C staining or on P6 for Iba1 immunohistochemistry. In adults, punch biopsies for qRT-PCR were taken from the medial superficial dorsal horn 3 d following adult incision.

In additional experiments, male and female P3 rat pups were randomly assigned to three experimental groups: neonatal DMSO vehicle (nv, *n* = 8), neonatal vehicle plus incision (nvIN, *n* = 8), and neonatal intrathecal SB203580 plus incision (nSBIN, *n* = 8 males and 8 females). Mechanical withdrawal thresholds were measured at baseline on P3, and then 4, 24, 48, and 72 h after neonatal intervention. At 7–8 weeks of age, mechanical thresholds were measured at baseline and at regular intervals to 21 d after left hindpaw incision (nv-IN, nvIN-IN, and nSBIN-IN). To evaluate potential tissue effects of repeat intrathecal SB203580 in 8% DMSO, spinal cords were collected for FJ-C staining following P3 injection of 0.4 mg/kg 24 h previously plus 0.3 mg/kg 6 h before death on P4.

To assess the anatomical distribution of reincision hyperalgesia, P3 incision was performed at 5 sites: left or right hindpaw (nIN ipsilateral or contralateral), left anterior thigh (nThi), left or right forepaw (nFor ipsilateral or contralateral) in P3 male rat pups. Littermate controls received the same duration of neonatal anesthesia, handling, and maternal separation. Mechanical and thermal hindlimb thresholds were measured in adulthood (7–8 weeks of age) and at regular intervals to 21 d after incision of the left hindpaw in all groups (nIN-IN ipsilateral or contralateral, nThi-IN, nForIN-IN ipsilateral or contralateral). As behavioral responses to hindpaw incision did not differ between males and females, and no additional intervention was performed, these experiments were performed only in males.

##### Statistical analysis.

Our primary outcome was the impact of neonatal minocycline on reincision hyperalgesia in adult male and female rats. Based on comparisons of sensory threshold in male and female adult rats from the same in-house colony using the same test protocol (Walker et al., 2015), a sample size of 8 was chosen (80% power at *p* < 0.01 for detecting a 20 and 25% difference in mechanical withdrawal threshold in males and females, respectively; 80% power at *p* < 0.05 for detecting a 35% difference in thermal withdrawal latency). Sensory threshold data are also presented as percentage of baseline [(postincision threshold)/preincision baseline threshold) × 100] plotted against time. To incorporate differences in both the degree and duration of hyperalgesia, the hyperalgesic index for each animal was calculated as the area over the percentage change sensory threshold versus time curve from baseline (0) to 21 d, such that a larger area over the curve represents a greater change from baseline and greater degree and/or duration of hyperalgesia. Based on our previous data (Beggs et al., 2012; Schwaller et al., 2015), a sample size of 8 has 90% power for detecting a 30% difference (*p* < 0.05) in mechanical hyperalgesic index following adult incision.

Behavioral data were normally distributed (D'Agostino and Pearson omnibus normality test) and analyzed by unpaired Student's *t* test (baseline thresholds) or three-way ANOVA with sex, incision, and drug (minocycline or saline) as factors. Data are graphed separately for males and females and analyzed with two-way ANOVA with group and sex as variables, and timeline data with repeated measures; time as the within-subjects factor and treatment group as between-subject factors. Dunnett's *post hoc* tests were used to assess changes relative to baseline and Bonferroni *post hoc* tests to assess between-group differences, with *p* values adjusted for multiple comparisons. Normalized RT-PCR data were analyzed by two-way ANOVA with sex and drug as factors, followed by Bonferroni *post hoc* tests as appropriate. Cell counts (Iba1, FJ-C) were analyzed by three-way ANOVA (sex, surgery, and drug) with Bonferroni *post hoc* tests.

For clarity of behavioral timelines, data points are represented as mean ± SEM. For other outcomes, individual data points are shown with bars representing mean ± SD. Data were analyzed with Prism (version 7, GraphPad, RRID:SCR_002798) or SPSS (IBM, version 22, RRID:SCR_002865). *p* < 0.05 was considered statistically significant; *p* values are reported in the text apart from very small values <0.001, which is designated as *p* < 0.001.

## Results

### Reincision hyperalgesia following neonatal incision is equivalent in adult males and females

**Figure 2.**
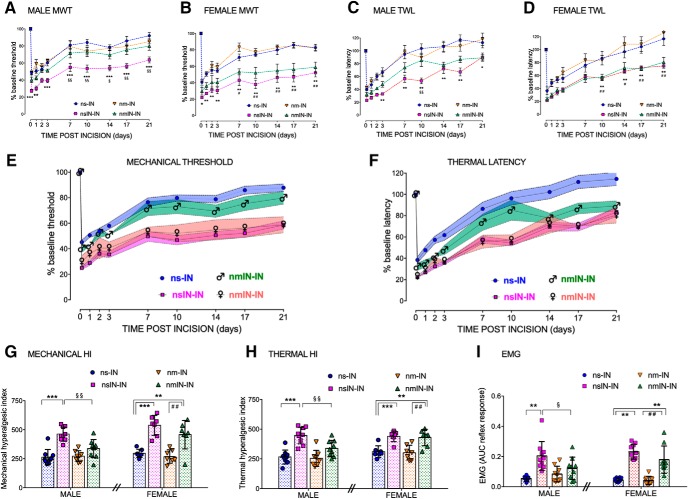
Mechanical and thermal hyperalgesia following adult reincision is sex-dependently influenced by minocycline at the time of neonatal incision. ***A–D***, Changes in behavioral thresholds following incision are normalized as percentage change from baseline (adult preincision). Data points are mean ± SEM. *n* = 8 or 9 animals per group analyzed by two-way repeated-measures ANOVA with Bonferroni *post hoc* comparisons. Mechanical withdrawal threshold (MWT) in male (***A***) and female (***B***) rats and thermal withdrawal latency (TWL) in male (***C***) and female (***D***) rats are plotted against time points to 21 d after incision. Hyperalgesia is enhanced by prior incision (nsIN-IN vs nsIN: ***p < 0.001; **p < 0.01; *p < 0.05) in both males and females. ***A***, In male rats, neonatal minocycline treatment significantly attenuated the enhanced mechanical hyperalgesia from 7 to 21 d (nmIN-IN vs nsIN-IN: ^§§^*p* < 0.01; ^§^*p* > 0.05). ***B***, In female rats, mechanical hyperalgesia following reincision was enhanced 7–21 d following incision despite neonatal minocycline (nm-IN vs nmIN-IN: ^##^*p* < 0.01; ^#^*p* < 0.05). Differences in thermal latency in males (***C***) and females (***D***) were less marked but followed the same overall pattern with neonatal minocycline attenuating reincision hyperalgesia in males but not females. ***E***, ***F***, Summary figures of mechanical (***E***) and thermal (***F***) hyperalgesia highlight sex-dependent differences following neonatal incision with minocycline (nmIN-IN). Within the ns-IN and nsIN-IN groups, data did not differ between males and females and are combined to minimize overlap in the figure. The impact of prior neonatal incision is highlighted by the clear separation in both the degree and duration of hyperalgesia (ns-IN vs nsIN-IN). In males, minocycline at the time of neonatal incision prevents adult reincision hyperalgesia (♂ nmIN-IN differs from nsIN-IN), and this group more closely approximates animals with no prior neonatal injury (ns-IN). In females, minocycline at the time of neonatal incision (♀ nmIN-IN) has no effect; enhanced adult reincision hyperalgesia persists, and this group approximates the nsIN-IN group. ***G***, ***H***, Behavioral data are expressed as the hyperalgesic index (area over the curve to 21 d after incision) of mechanical threshold (***G***) or thermal latency (***H***). Reincision hyperalgesia is apparent in males and females (nsIN vs nsIN-IN: ***p < 0.001; **p < 0.01) and is reduced by neonatal minocycline in males only (nsIN-IN vs nmIN-IN: ^§§^*p* < 0.01). In females, enhanced reincision hyperalgesia persists despite minocycline (nmIN vs nmIN-IN: ^##^*p* < 0.01). Data points are individual animals (*n* = 8 or 9 per group). Error bars indicate mean ± SD analyzed by two-way ANOVA with Bonferroni *post hoc* comparisons. ***I***, Reflex sensitivity 24 h following adult incision quantified as the area under the stimulus (hindpaw mechanical von Frey hair) versus response curve (electromyography recording biceps femoris; EMG area under curve reflex response) demonstrates reincision hyperalgesia in males and females (nsIN-IN>ns-IN: **p < 0.01). Neonatal minocycline (nmIN-IN) reduced the reincision response in males only (nmIN-IN < nsIN-IN: ^§^*p* < 0.05), but enhanced hyperalgesia persisted in females (nmIN-IN > nsIN: **p < 0.001). Data points are individual animals (*n* = 9 or 10 per group). Error bars indicate mean ± SD analyzed by two-way ANOVA with sex and group as variables and Bonferroni *post hoc* comparisons. Groups include the following: ns-IN, nm-IN, nsIN-IN, and nmIN-IN.

To support our previous finding of enhanced reincision hyperalgesia following neonatal incision (Beggs et al., 2012; Schwaller et al., 2015), larger groups of males and females were compared, to evaluate potential sex differences in behavioral response. Mechanical withdrawal thresholds following adult incision (nsIN-IN vs ns-IN) were influenced by prior neonatal incision (*F*_(1,28)_ = 11.2, *p* = 0.002) but not sex (*F*_(1,28)_ = 1.7, *p* = 0.20); and similarly, thermal withdrawal latency was influenced by prior neonatal incision (*F*_(1,28)_ = 6.1, *p* = 0.02) but not sex (*F*_(1,28)_ = 0.7, *p* = 0.41). Differences in the degree of hyperalgesia following adult incision were not solely due to alterations in adult baseline values, as nsIN-IN groups had both higher preincision and lower postincision thresholds for mechanical withdrawal threshold (nsIN-IN vs nsIN: baseline mean ± SD 30.2 ± 5.4 vs 24.9 ± 3.1 g; *t*_(29)_ = 3.3, *p* = 0.002; 4 h after incision, 7.4 ± 1.4 vs 11.1 ± 1.3 g; *t*_(29)_ = 7.6, *p* < 0.001). Differences in thermal latency were less marked (nsIN-IN vs nsIN: baseline 12.1 ± 2.1 vs 10.8 ± 1.8 s *t*_(29)_ = 1.9, *p* = 0.056; 4 h after incision, 3.3 ± 1.1 vs 4.1 ± 1.1 s; *t*_(29)_ = 2.0, *p* = 0.052). Expression as percentage change from baseline facilitated comparison of the relative change across groups and demonstrated increased hyperalgesia following neonatal incision at time points to 21 d in males and females (nsIN-IN vs nsIN; Fig. 2*A–D*). Prior neonatal incision increases mechanical and thermal behavioral hyperalgesia to an equivalent degree in males and females.

### Neonatal perioperative minocycline prevents enhanced reincision hyperalgesia in adult males but not females

We next evaluated the potential for a neonatal intervention to prevent long-term alterations in injury response. While there was a significant main effect of prior incision in both males (*F*_(1,31)_ = 33.3, *p* < 0.001) and females (*F*_(1,24)_ = 48, *p* < 0.001), minocycline had a significant effect in males (*F*_(1,31)_ = 6.6, *p* = 0.02) but not females (*F*_(1,24)_ = 2.8, *p* = 0.10). In males, neonatal mincocycline prevented reincision mechanical hyperalgesia (nmIN-IN did not differ from ns-IN) and significant differences between nsIN-IN and nmIN-IN groups emerged after 7 d (Fig. 2*A*). In females, mechanical hyperalgesia did not differ from the reincision saline group (nmIN-IN vs nsIN-IN) and enhanced hyperalgesia persisted (nmIN-IN vs ns-IN from 7 d after incision; Fig. 2*B*). Thermal withdrawal latencies similarly showed a main effect of prior incision in both males (*F*_(1,31)_ = 38.5, *p* < 0.01) and females (*F*_(1,24)_ = 27.0, *p* < 0.001). At time points after 10 d, thermal latency in the male nmIN-IN group differed from nsIN-IN (i.e., reduced reincision hyperalgesia; Fig. 2*C*), whereas in females nmIN-IN differed from the ns-IN group (i.e., reincision hyperalgesia persisted; Fig. 2*D*). Summary figures for mechanical threshold (Fig. 2*E*) and thermal latency (Fig. 2*F*) highlight the differences in male and female nmIN-IN groups, with sex-dependent differences particularly apparent 7–10 d after adult reincision.

Neonatal minocycline alone, in the absence of neonatal incision (nm), did not alter the response to adult incision in males or females. The degree and duration of incision-induced hyperalgesia in adulthood did not differ between neonatal minocycline and neonatal saline control groups (nm-IN vs ns-IN; Fig. 2*A–D*).

To provide a composite measure encompassing both the degree and duration of behavioral response, hyperalgesic indices (area over threshold vs time 0–21 d) were calculated. For mechanical hyperalgesic index, there were significant main effects of incision (*F*_(1,55)_ = 88.1; *p* < 0.001), sex (*F*_(1,55)_ = 9.3, *p* = 0.003), and drug (*F*_(1,55)_ = 9.4, *p* = 0.003). Similarly, thermal hyperalgesic index showed a main effect of incision (*F*_(1,55)_ = 68.1, *p* < 0.001), sex (*F*_(1,55)_ = 7.7, *p* = 0.007), and drug (*F*_(1,55)_ = 4.6, *p* = 0.036) (three-way factorial ANOVA). Reincision mechanical hyperalgesia was modulated by neonatal minocycline in males (nmIN-IN < nsIN-IN, *p* = 0.002), but in females an enhanced response persisted (nmIN-IN > ns-IN, *p* = 0.008 and nmIN-IN > nm-IN, *p* = 0.002) (Fig. 2*G*). Similar results were seen with thermal hyperalgesic index in males (nmIN-IN < nsIN-IN, *p* = 0.009) and females (nmIN-IN > ns-IN, *p* = 0.004 and nmIN-IN > nm-IN, *p* = 0.002) (Fig. 2*H*).

To confirm that differences were not restricted to behavioral withdrawal thresholds, reflex sensitivity to threshold and suprathreshold stimuli was quantified by electromyographic recordings in anesthetized animals 24 h following adult incision. There were significant main effects of incision (*F*_(1,68)_ = 87.6, *p* < 0.001) and interactions between incision and sex (*F*_(1,68)_ = 5.8, *p* = 0.004) and incision and drug (*F*_(1,68)_ = 8.8, *p* = 0.019). Minocycline at the time of neonatal incision (nmIN-IN) reduced reincision hyperalgesia in males (nmIN-IN < nsIN-IN, *p* = 0.024), but in females reflex sensitivity was enhanced (nmIN-IN > ns-IN, *p* < 0.001) (Fig. 2*I*).

These data demonstrate that, while reflex sensitivity is enhanced by prior neonatal incision in both males and females, administering minocycline at the time of neonatal injury prevents the long-term reincision hyperalgesia in males only, and the same dose is ineffective in females.

### Adult incision increases spinal cord microglial-specific gene expression, but modulation by neonatal minocycline is sex-dependent

**Figure 3.**
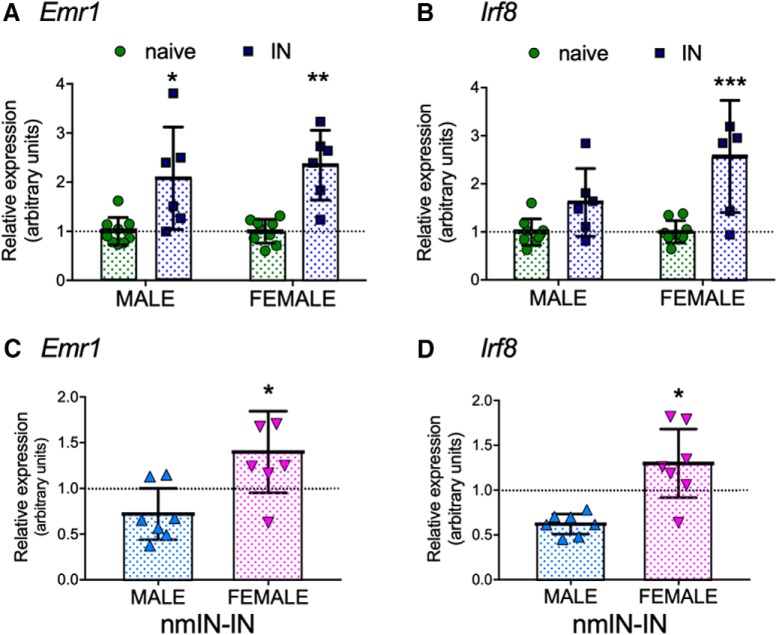
Incision, neonatal minocycline, and sex influence expression of microglial-related genes in the medial ipsilateral dorsal horn. ***A***, Expression of *Emr1* increased 3 d following single adult incision (IN) in males and females. ***B***, Expression of *Irf8* increased following IN. ***A***, ***B***, Data normalized to age- and sex-matched naive rats. ***C***, Expression of *Emr1* was lower in males than females following neonatal minocycline and reincision (nmIN-IN). Male nmIN-IN versus female nmIN-IN: *p* = 0.023. ***D***, Expression of *Irf8* was lower in males than females following neonatal minocycline and reincision (male nmIN-IN vs female nmIN-IN: *p* = 0.019). ***C***, ***D***, nmIN-IN data normalized to saline repeat incision (nsIN-IN). ***A–D***, Data points are individual units with each including 2 animals (*n* = 6–9 units per group). Error bars indicate ± SD. *p < 0.05; **p < 0.01; ***p < 0.001; two-way ANOVA with sex and group as variables and Bonferroni *post hoc* comparisons.

As spinal expression of genes associated with microglial proliferation (*Emr1*) and transformation to a reactive phenotype (*Irf8*) increase following peripheral nerve injury in adult rodents (Masuda et al., 2012; Sorge et al., 2015), we first determined whether expression of these genes was also increased in the medial superficial dorsal horn following hindpaw incision in adult rodents. Expression was assessed 3 d following incision, as spinal microglial reactivity (Iba1 immunohistochemistry) increased at this time point in adults without prior neonatal injury (Beggs et al., 2012). *Emr1* expression showed a main effect of incision (*F*_(1,26)_ = 30.4, *p* < 0.001) but not of sex (*F*_(1,26)_ = 0.4, *p* = 0.55) (Fig. 3*A*). Similarly, there was a main effect of incision (*F*_(1,26)_ = 21.5, *p* < 0.001) but not sex (*F*_(1,26)_ = 4.1, *p* = 0.053) on *Irf8* expression (two-way ANOVA; Fig. 3*B*).

As prior neonatal incision alters the time course, degree, and distribution of spinal microglial response (Beggs et al., 2012; Schwaller et al., 2015), effects of neonatal minocycline following adult incision (nmIN-IN) were normalized against the reincision saline group (nsIN-IN). There were significant effects of sex and sex × drug interactions for both *Emr1* (*F*_(1,27)_ = 5.5, *p* = 0.027) and for *Irf8* (*F*_(1,27)_ = 5.7, *p* = 0.024). In neonatal minocycline groups, expression following adult reincision (nmIN-IN) was significantly lower in males than in females for *Emr1* (*p* = 0.023; Fig. 3*C*) and *Irf8* (*p* = 0.019; Fig. 3*D*). Therefore, in addition to sex-dependent long-term effects on behavioral hyperalgesia, neonatal minocycline specifically affects the spinal microglial response following reincision, but in males only.

### Neonatal intrathecal p38 inhibitor prevents enhanced reincision mechanical hyperalgesia in adult males but not females

**Figure 4.**
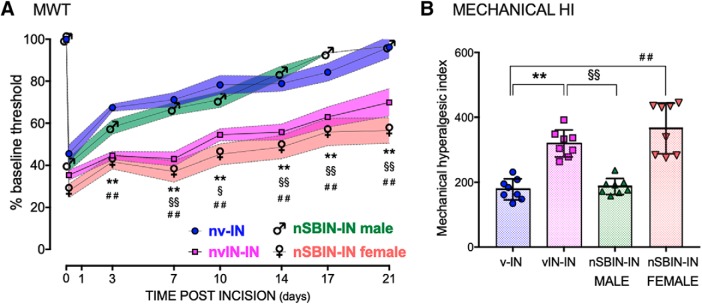
Mechanical hyperalgesia following adult reincision is sex-dependently influenced by intrathecal SB203580 at the time of neonatal incision. ***A***, Changes in mechanical withdrawal threshold following incision are normalized as percentage change from baseline (adult preincision). Within the nv-IN and nvIN-IN groups, data did not differ between males and females, and are combined. The impact of prior neonatal incision is highlighted by the clear separation in both the degree and duration of hyperalgesia (nv-IN vs nvIN-IN: **p < 0.001). In males, SB203580 at the time of neonatal incision attenuated the enhanced reincision mechanical hyperalgesia from 7 to 21 d (♂ nSBIN-IN vs nvIN-IN: ^§§^*p* < 0.001; ^§^*p* > 0.05). In females, neonatal SB203850 has no effect; enhanced adult reincision hyperalgesia persists 3–21 d following incision (♀ nSBIN-IN vs nv-IN: ^##^*p* < 0.001). Data points are mean ± SEM, analyzed by two-way repeated-measures ANOVA with Bonferroni *post hoc* comparisons. *n* = 8 animals per group. ***B***, The mechanical hyperalgesic index (area over the curve 0–21 d after incision) identifies enhanced reincision hyperalgesia (nv-IN vs nvIN-IN: **p < 0.001) that is reduced by neonatal SB203580 in males only (nSBIN-IN males vs nvIN-IN: ^§§^*p* < 0.01). In females, enhanced hyperalgesia persists (nSBIN-IN female vs nv-IN: ^##^*p* < 0.01). Data points are individual animals (*n* = 8 per group). Error bars indicate mean ± SD, analyzed by two-way ANOVA with Bonferroni *post hoc* comparisons.

As microglial P2X_4_ receptors are a key point of divergence for sex-dependent responses in neuroglial signaling in adult rodents (Mapplebeck et al., 2018), we also evaluated the effect of inhibition of the downstream p38 MAPK signaling pathway. Prior neonatal incision increased adult-incision induced expression of microglial phospho-p38 and anti-allodynic efficacy of the p38 MAPK inhibitor SB203580 (Schwaller et al., 2015). Here, SB203580 was administered intrathecally at the time of neonatal incision (nSBIN; 1 mg/kg in divided doses 30 min before and 24 and 48 h after incision). In vehicle control animals, prior neonatal incision was again associated with higher baseline mechanical withdrawal threshold in adulthood in both the ipsilateral (nvIN vs nv; 29.3 ± 3.6 vs 24.1 ± 2.2 g; *t* (14) = 3.5, *p* = 0.010) and contralateral paw, and an increased degree and duration of reincision hyperalgesia. Changes in mechanical withdrawal threshold following adult incision (nvIN-IN vs nv-IN) were influenced by time (*F*_(7,84)_ = 47, *p* < 0.001) and prior neonatal incision (*F*_(1,12)_ = 36, *p* < 0.001). As there was no main effect of sex (*F*_(1,12)_ = 0.48, *p* = 0.5) or sex × group interaction (*F*_(1,12)_ = 0.3, *p* = 0.6), male and female data were combined in subsequent analyses of nv-IN and nvIN-IN groups (Fig. 4).

Following neonatal incision with intrathecal SB203580 (nSBIN), baseline mechanical withdrawal thresholds in adulthood were higher and more variable in females than males (35.1 ± 8.9 g vs 25.3 ± 1.2 g; *t* (14) = 3.1, *p* = 0.01). Despite this higher baseline, raw mechanical withdrawal thresholds following reincision (nSBIN-IN) were significantly lower in females than males at time points from 7 to 21 d after incision (*p* < 0.05; two-way repeated-measures ANOVA with Bonferroni *post hoc* comparisons). Expression as percentage change from baseline facilitated comparison across all groups (nv-IN vs nvIN-IN vs nSBIN-IN males vs nSBIN-IN females; *n* = 8 per group; Fig. 4). There was a significant main effect of group (*F*_(3,28)_ = 29, *p* < 0.001) with differences between male and female nSBIN-IN groups initially at 3 d (*p* = 0.02) that were more marked from 7 to 21 d after incision (*p* < 0.001; two-way repeated-measures ANOVA with Bonferroni *post hoc* comparisons; Fig. 4*A*). Neonatal microglial inhibition with intrathecal SB203580 prevented reincision hyperalgesia in males (nSBIN-IN males vs nvIN-IN, *p* < 0.001), and this group did not differ from adults without prior incision (nsSBN-IN males vs nv-IN, *p* = 0.9). By contrast, in females, reincision hyperalgesia was evident (nSBIN-IN females vs nv-IN, *p* < 0.001), and values did not differ from the vehicle reincision group (nSBIN-IN females vs nvIN-IN, *p* = 0.4).

The composite measure of mechanical hyperalgesic index (0–21 d) similarly highlighted a main effect of prior neonatal incision (*F*_(1,26)_ = 27, *p* < 0.001), sex (*F*_(1,26)_ = 14, *p* = 0.002), and sex × drug interaction (*F*_(1,26)_ = 13, *p* = 0.001). Enhanced reincision hyperalgesia (nv-IN vs nvIN-IN, *p* < 0.001) was prevented by neonatal SB203580 in males (nSBIN-IN males vs nvIN-IN, *p* < 0.001) but was still evident in females (nSBIN-IN females vs nv-IN, *p* < 0.001) (Fig. 4*B*).

### Enhanced hyperalgesia is not restricted to reincision of the same paw

**Figure 5.**
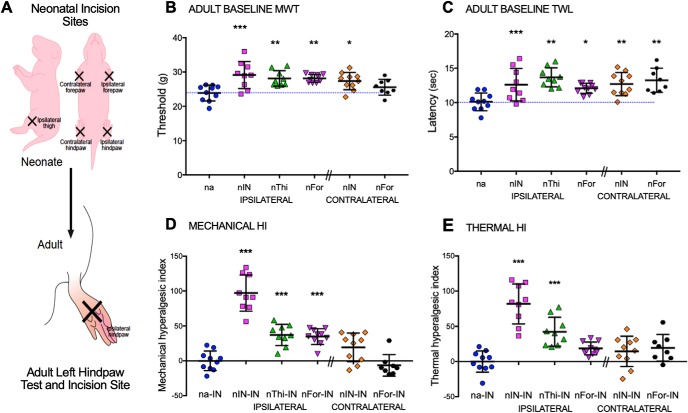
Distribution of baseline hypoalgesia and reincision hyperalgesia in adults differs following neonatal incision. ***A***, The schematic demonstrates different ipsilateral or contralateral incision sites (nIN, hindpaw; nThi, thigh; nFor, forepaw) performed in neonatal (P3) animals, which are followed by incision of the left hindpaw in adulthood. ***B***, Mechanical withdrawal thresholds of the left hindpaw in young adult rats were higher than neonatal anesthesia (na) controls following neonatal incision of the ipsilateral hindpaw (*p* < 0.001), thigh (*p* = 0.003), forepaw (nFor *p* = 0.002), and contralateral hindpaw (*p* = 0.015). ***C***, Thermal withdrawal latency of the left hindpaw was prolonged following prior incision at all sites (na vs nIN *p* = 0.006, vs nThi *p* < 0.001, vs nFor *p* = 0.033, vs contralateral nIN *p* = 0.003, vs contralateral nFor *p* = 0.001). ***D***, Mechanical hyperalgesic index (HI) following adult incision (area over behavioral withdrawal curve vs time to 21 d after incision) was increased by prior ipsilateral incision. ***E***, Thermal HI was similarly increased following prior ipsilateral hindpaw or thigh incision, but not contralateral hindpaw incision. Forepaw incisions did not significantly alter thermal HI. ***B–E***, Data are presented for individual animals (*n* = 8–10 per group). Bars indicate mean ± SD. ***p < 0.001; **p < 0.01; *p < 0.05; one-way ANOVA with Dunnett's test, compared with neonatal anesthesia (na: ***B***, ***C***) or neonatal anesthesia plus adult incision (na-IN: ***D***, ***E***).

Neonatal incision produces baseline hypoalgesia and reincision unmasks hyperalgesia in adulthood. We have previously shown that elevated baseline thresholds have a generalized distribution, with enhanced descending inhibition from the rostroventral medulla influencing reflex sensitivity regardless of prior incision on the ipsilateral or contralateral hindpaw or forepaw (Walker et al., 2015). Hindpaw carrageenan inflammation in the first postnatal week, but not at older ages, is similarly associated with generalized hypoalgesia in adulthood, whereas an enhanced hyperalgesic response is restricted to reinflammation of the same, but not contralateral, hindpaw (Ren et al., 2004). As we have previously assessed reincision in the same paw only, we now evaluated the degree and distribution of hyperalgesia following neonatal incision at different body sites. The same length of initial incision was performed either on the left or right hindpaw (nIN ipsilateral or contralateral), left anterior thigh (nThi), left or right forepaw (nFor ipsilateral or contralateral) at P3, and we have previously shown that forepaw and hindpaw incisions produce similar acute hyperalgesia at this age (Walker et al., 2015). Hindlimb reflex thresholds were then assessed at baseline and following incision of the left hindpaw in adulthood (Fig. 5). As our previous experiments had shown no difference in behavioral response in males and females, these experiments were performed in males only.

At 6–7 weeks of age, baseline mechanical withdrawal threshold in the left hindpaw was significantly altered following prior neonatal incision (main effect of group *F*_(5,50)_ = 5.7, *p* < 0.001) with thresholds higher following prior incision in all sites, apart from the contralateral forepaw; Fig. 5*A*). Thermal latency was increased following all prior neonatal incisions with a main effect of group (*F*_(5,50)_ = 5.9, *p* < 0.001; Fig. 5*B*).

In all adults, the left hindpaw was incised to facilitate comparison across groups (Fig. 5). Mechanical withdrawal thresholds and thermal latency were plotted against time and expressed as percentage change from baseline (data not shown) for calculation of hyperalgesic indices. The mechanical hyperalgesic index (0–21 d) was increased following prior ipsilateral (na-IN vs nIN-IN, nThi, or nFor-IN; all *p* < 0.001) but not contralateral incision (contralateral hindpaw nIN-IN, *p* = 0.07; contralateral forepaw nFor-IN, *p* = 0.9) (one-way ANOVA with Dunnett's comparison to na-IN). Thermal data demonstrated enhanced hyperalgesia following prior incision of the same paw and ipsilateral thigh (na-IN vs nIN-IN or nThi, *p* < 0.001), but not ipsilateral forepaw (*p* = 0.14) or contralateral hindpaw (*p* = 0.35) or forepaw (*p* = 0.16). The relative changes in hyperalgesic index highlight that increased mechanical hyperalgesia was maximal with reincision in the same paw (nIN-IN vs IN, mean ± SD: 97 ± 25% increase), but also increased following prior ipsilateral anterior thigh (37 ± 15%) or ipsilateral forepaw (35 ± 11%) incision (Fig. 5*C*). Similarly, thermal hyperalgesia was enhanced following prior ipsilateral incision, with maximal effect when the same hindpaw was incised (82 ± 28%), but prior contralateral hindpaw incision had no effect (Fig. 5*D*). In adults with prior neonatal incision, we have previously shown that enhanced hyperalgesia and spinal microglial reactivity are independent of peripheral reinjury and can be induced by lateral thigh incision and tibial nerve stimulation (Beggs et al., 2012), and these current data further support a role for segmentally restricted spinal mechanisms in the primed response to injury following neonatal surgical incision.

### Neonatal incision produces acute hyperalgesia and a spinal microglial response in male and female rat pups

**Figure 6.**
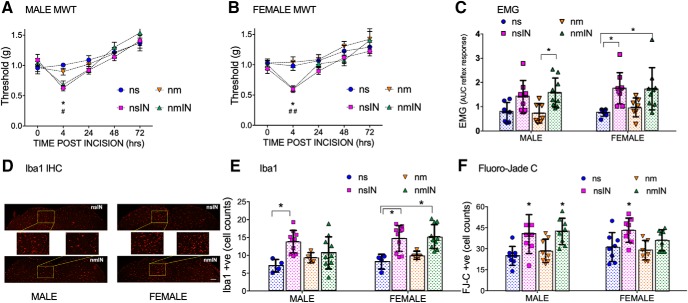
Acute neonatal effects of incision and/or minocycline. ***A***, ***B***, Mechanical withdrawal threshold (MWT) is reduced 4 h following neonatal saline and incision (nsIN) in male (***A***) and female (***B***) rat pups compared with nonincised saline (ns) and minocycline (nm) controls. Minocycline at the time of neonatal incision (nmIN) has no effect. Data are mean ± SEM (*n* = 6 ns animals, *n* = 10 all other groups). ns vs nsIN: *p < 0.05; ns vs nmIN: ^##^*p* < 0.01; ^#^*p* < 0.05; two-way repeated-measures ANOVA with Bonferroni *post hoc* comparisons. ***C***, Twenty-four hours following P3 interventions, reflex sensitivity was quantified as the area under the curve of the stimulus (von Frey hair to hindpaw) versus biceps femoris EMG response. Data points represent individual animals (*n* = 7–9 per group). Error bars indicate mean ± SD. Male nmIN > ns, *p* = 0.043; nmIN> nm, *p* = 0.035; female nsIN > ns, *p* = 0.007; nmIN> ns, *p* = 0.008; two-way ANOVA with Bonferroni *post hoc* comparisons. ***D***, Representative low- and high-power images of the dorsal horn of male and female rats 3 d following incision with perioperative saline (nsIN) or minocycline (nmIN). Scale bar, 210 μm. ***E***, Iba1-positive cells within a fixed ROI in the medial superficial dorsal horn were significantly increased following incision in males (ns vs nsIN, *p* = 0.007), and females given saline (ns vs nsIN, *p* = 0.011) or minocycline (ns vs nmIN, *p* = 0.005). Data points represent average of at least 6 spinal L4/5 sections for each individual animal (*n* = 4 ns or nm; *n* = 10 nsIN or nmIN). ***F***, FJ-C-positive cell counts in the ipsilateral (left) lumbar cord (L4,5 segments) were increased following incision in males (ns vs nsIN, *p* = 0.008; ns vs nmIN, *p* = 0.002; nm vs nmIN, *p* = 0.024) and females (nm vs nsIN, *p* = 0.019). Data points represent sum of FJ-C-positive counts from 6 L4/5 spinal cord sections per animal (*n* = 8 animals per group). ***E***, ***F***, Error bars indicate SD. *p < 0.05, two-way ANOVA with Bonferroni *post hoc* comparisons. Groups are as follows ns, nm, nsIN, and nmIN.

To evaluate acute effects of microglial inhibition and incision, we also present data from the neonatal period. Plantar incision at P3 acutely reduced mechanical withdrawal threshold with lower mechanical withdrawal threshold 4 h after incision in males (nsIN < ns, *p* = 0.011; nmIN < ns, *p* = 0.045; Fig. 6*A*) and females (nsIN < ns, *p* = 0.046; nmIN < ns, *p* = 0.003; two-way repeated measures with Bonferroni *post hoc* comparisons; Fig. 6*B*). Withdrawal thresholds in nonincised saline and minocycline groups did not differ at any time point. Reflex sensitivity to both threshold and more intense suprathreshold mechanical stimuli (quantified by EMG response 24 h after P3 incision) showed a main effect of treatment group (*F*_(3,55)_ = 10.4, *p* < 0.001), but not sex (*F*_(1,55)_ = 1.6, *p* = 0.21) (two-way ANOVA with sex and group as variables; Fig. 6*C*). Minocycline did not prevent acute hyperalgesia in incised rats, and values did not differ between minocycline alone and saline controls (Fig. 6*A–C*). This suggests that effects of systemic minocycline are not due to the nonspecific acute peripheral anti-inflammatory effects shown with higher doses of systemic minocycline in adult animals (Beggs et al., 2012).

Four hours following neonatal incision, mechanical withdrawal thresholds were reduced from baseline in intrathecal vehicle (nvIN, *p* = 0.04) and female SB203580 groups (nSBIN, *p* = 0.04) but to a reduced degree in males (nSBIN males, *p* = 0.38; two-way repeated measures with Bonferroni *post hoc* comparisons). Overall, values did not differ significantly across groups at each time point, and the normal developmental increase in mechanical withdrawal threshold with postnatal age was evident (P6 > P3 baseline, all groups, *p* < 0.001).

Three days following neonatal incision, analysis of the number of Iba1-positive cells in the medial superficial ipsilateral dorsal horn (Fig. 6*D*) demonstrated a main effect of incision (*F*_(1,48)_ = 25.3, *p* < 0.001) but not sex (*F*_(1,48)_ = 3.4, *p* = 0.07) or drug (*F*_(1,48)_ = 0.01, *p* = 0.72). Minocycline did not prevent incision-induced increases in Iba1-positive cell counts in males or females, although analysis was limited by variability in this outcome (Fig. 6*E*).

In neonatal rodents, normal developmental neuronal apoptosis occurs predominantly in the dorsal horn of the spinal cord (Lowrie and Lawson, 2000) but can be increased by injury and anesthesia/analgesia (Walker and Yaksh, 2012; Chiarotto et al., 2014); and in the developing brain, systemic minocycline has been reported to paradoxically increase brain cell death in an age-dependent (Arnoux et al., 2014) and dose-dependent manner (5-fold increase in somatosensory cortex following 5 × 45 mg/kg between P3 and P5) (Strahan et al., 2017). Therefore, we used FJ-C staining to assess cell death 24 h following P3 interventions. At P4, FJ-C counts were higher in the dorsal versus ventral horn (ns 28 ± 7 vs 9 ± 4; mean ± SD, summed from 6 sections per animal). In the ipsilateral dorsal horn, FJ-C cell counts showed a main effect of incision (*F*_(1,56)_ = 26.7, *p* < 0.001) but not sex (*F*_(1,56)_ = 0.13, *p* = 0.72) or minocycline administration (*F*_(1,56)_ = 0.18, *p* = 0.68) (Fig. 6*F*). FJ-C cell counts increased 24 h following incision in males (nsIN vs ns: 41 ± 14 vs 25 ± 7) and females (nsIN vs ns: 43 ± 9 vs 31 ± 10). This relative increase (40%–60%) was lower than following intrathecal ketamine doses at P3 (>300% increase) that were also associated with long-term alterations in adult hindlimb sensory thresholds and gait (Walker et al., 2010). Dorsal horn FJ-C counts following two intrathecal doses of SB203580 in 8% DMSO did not significantly differ from saline or minocycline nonincision groups (nSB vs ns vs nm: 33 ± 5 vs 28 ± 7 vs 29 ± 7, *p* = 0.36). Therefore, the pharmacological interventions used here did not cause paradoxical cell death in the neonatal spinal cord.

To exclude effects of injury or minocycline on growth and sensorimotor function, body weight and spontaneous locomotor activity were measured in adulthood. Males were heavier than females (mean ± SD: 312 ± 17 g vs 218 ± 21 g; *t*_(59)_ = 19.2, *p* < 0.001), but within sexes, weight did not differ markedly across treatment groups (data not shown). Spontaneous locomotor activity was assessed by distance traveled during 3 min in a 90 × 90 cm open field 24 h following adult incision. Males were less active than females (distance traveled mean ± SD: 11.9 ± 3.8 m vs 14.1 ± 4.8 m) resulting in a main effect of sex (*F*_(1,64)_ = 6.14, 0.016), but there was no effect of incision (*F*_(1,64)_ = 0.06, *p* = 0.81) or minocycline (*F*_(1,64)_ = 2.71, *p* = 0.81) on distance traveled.

## Discussion

Prior neonatal incision has a long-term impact on somatosensory processing, and enhanced postsurgical hyperalgesia following adult reincision is abolished by microglial inhibitors in adult males (Beggs et al., 2012; Schwaller et al., 2015). We now demonstrate persistent sexually dimorphic effects following microglial inhibition in early development: neonatal peri-incision minocycline prevents reincision hyperalgesia only in adult males, and dorsal horn genes related to microglial function are downregulated in males but upregulated in females. MAPK signaling is involved as neonatal intrathecal SB203850 also prevented reincision hyperalgesia in males only. Following neonatal incision at different sites, adult reincision hyperalgesia is restricted to prior ipsilateral injury and is maximal when the same paw is reinjured, supporting a segmentally restricted spinal mechanism.

Hindpaw incision produces equivalent acute hyperalgesia in male and female rat pups, and enhanced hyperalgesia following subsequent adult reincision is also independent of sex. In adults, spinal microglial inhibition selectively minimized reincision hyperalgesia at doses that were ineffective following adult-only incision (Beggs et al., 2012; Schwaller et al., 2015), but experiments were predominantly in males. Sexually dimorphic responses to microglial inhibition in adult rodents follow peripheral nerve injury and inflammation (Sorge et al., 2015; Mapplebeck et al., 2018), hindpaw formalin (Taves et al., 2016), and hyperalgesic priming to prostaglandin E_2_ (Paige et al., 2018). Here, the key finding is that microglial inhibition with systemic minocycline or intrathecal SB203580 at the time of neonatal injury has a long-term preventive effect: modulating reincision hyperalgesia in males only, with significant sex-dependent group differences following adult incision. These data suggest that the transition from acute to persistent postincision pain state is mediated by different mechanisms (Echeverry et al., 2017; Price et al., 2018) and more effectively modulated by neonatal microglial inhibition in males.

Sex-dependent responses to microglial inhibition following tissue injury in adult rodents are spinally mediated (Taves et al., 2016; Mapplebeck et al., 2018). While intrathecal LPSs induced mechanical allodynia only in adult males, intracerebroventicular or intraplantar LPSs produced equivalent allodynia in both sexes (Sorge et al., 2011). However, there has been limited evaluation of age-dependent changes in microglial function in the spinal cord. Compared with brain microglia, spinal microglia have a reduced *in vitro* inflammatory response to LPSs (Baskar Jesudasan et al., 2014). Behavioral and microglial responses in juvenile rodents also vary with type of injury. In male P10 rats, intrathecal LPS, but not spared nerve injury, produced acute hyperalgesia and increased spinal microgliosis, and age-dependent shifts between anti-inflammatory and proinflammatory spinal microglial responses influenced the delayed emergence of behavioral allodynia following nerve injury (Moss et al., 2007; McKelvey et al., 2015). Plantar incision induces microgliosis in the ipsilateral dorsal horn in both neonatal and adult rats, and prior incision increases the degree, duration, and distribution of the adult response (Beggs et al., 2012). To investigate potential sex differences in the underlying molecular pathway, we first confirmed that hindpaw incision upregulated *Emr1*, a marker of microglial proliferation, and *Irf8*, a transcription factor critical for adoption of a reactive phenotype (Masuda et al., 2012) in the ipsilateral dorsal horn of adult males and females. The response following neonatal microglial inhibition in the reincision groups was sex-dependent, with *Emr1* and *Irf8* upregulated in minocycline-treated females but downregulated in males. Spinal P2X_4_R-signaling pathways underlie sexually dimorphic effects to microglial inhibitors in adults (Sorge et al., 2015; Taves et al., 2016; Mapplebeck et al., 2018), and we now demonstrate a role for downstream MAPK signaling in the long-term preventive effects following neonatal incision in males, but not females.

Directly activating spinal microglia by intrathecal LPSs produces testosterone-dependent allodynia in male, but not female, mice (Sorge et al., 2011), and manipulating sex hormone levels also alters efficacy of microglial inhibitors, which become ineffective in castrated males (Sorge et al., 2015). Microglia in the developing and adult mouse brain show sex-specific transcriptomic and proteomic differences that can be influenced by, or be independent of, circulating sex hormones (Hanamsagar et al., 2017; Guneykaya et al., 2018; Nelson et al., 2018; Villa et al., 2018). The molecular mechanisms underlying this sex dichotomy are not well established (Villa et al., 2018); and following brain injury, the response to microglial inhibitors varies across studies. Minocycline efficacy following traumatic injury at P11 varied across brain regions, but sex had minimal impact (Hanlon et al., 2017). While minocycline improved outcome following brain hypoxia/ischemia in adult males only (Spychala et al., 2017), benefit in P3 rats has not been separately assessed in males and females (Wixey et al., 2009, 2011). Using a similar repeat dose regimen, which was well tolerated and improved outcome in rat pups, minocycline alone did not alter spinal reflex sensitivity or cell death in male or female rat pups. Peri-incision minocycline did not block acute injury-induced microgliosis or hyperalgesia following neonatal incision, suggesting that doses were insufficient to produce antihyperalgesic effects seen with high systemic doses in adults (Beggs et al., 2012). Nevertheless, systemic minocycline modulated priming by neonatal incision, producing long-term preventive effects on reincision hyperalgesia in adult males, but not females; and more selective inhibition with intrathecal SB203580 produced the same sexually dimorphic effects.

Priming of microglial responses may reflect intrinsic phenotypic changes with exaggerated responses to subsequent challenges (Perry and Holmes, 2014; Burke et al., 2016), and perinatal insults can alter the normal sex-dependent trajectory of microglial development (Hanamsagar et al., 2017). Microglia have multiple roles in normal activity-dependent refinement of sensory system circuitry (Salter and Stevens, 2017). Induction of long-term changes in reincision response is both developmentally regulated and activity-dependent. Blocking primary afferent input at the time of neonatal incision prevents early alterations in glutamatergic signaling (Li et al., 2009) and subsequent reincision hyperalgesia (Walker et al., 2009a; Moriarty et al., 2018). Neonatal plantar incision produces long-term increased gain in spinal nociceptive circuitry (Li et al., 2015; Li and Baccei, 2018), including increased monosynaptic input from low-threshold mechanoreceptors onto spinal projection neurons (Li and Baccei, 2016). As postnatal refinement of A-fiber distribution (Beggs et al., 2002) and maturation of local inhibitory circuitry in the spinal dorsal horn (Baccei and Fitzgerald, 2004; Koch et al., 2012) are sensitive to altered afferent input, microglial involvement in developing normal sensory circuits may also be influenced by injury-induced alterations in microglial reactivity.

A key consideration is whether priming by neonatal incision is dependent upon both neonatal and adult surgeries being performed at the same site. Adult incision rapidly induced extracellular signal-related kinase phosphorylation in spinal dorsal horn neurons, but the distribution and degree were not influenced by prior neonatal incision (Schwaller et al., 2015). Effects are also not dependent on peripheral reinjury, as tibial nerve electrical stimulation increased the degree of hyperalgesia and microglial reactivity in adults with prior neonatal incision. Microglial reactivity extended beyond the somatotopic afferent field of the initial hindpaw injury, with enhanced microgliosis related to the ipsilateral mid-thigh incision required to expose the nerve (Hathway et al., 2009; Beggs et al., 2012), and microglial phospho-p38 expression in adults with prior neonatal incision was also more extensive (Schwaller et al., 2015). While enhanced hyperalgesia was greatest when neonatal and adult incisions were at the same location, behavioral responses to adult hindpaw incision were also primed following neonatal incision at other ipsilateral but not contralateral sites, supporting a segmental mechanism that differs from effects on baseline threshold. Following neonatal hindpaw incision or inflammation, baseline hypoalgesia emerges after the fourth postnatal week and is generalized to ipsilateral and contralateral paws in adulthood (Ren et al., 2004; Walker et al., 2015; Moriarty et al., 2018). We hypothesize that these phenomena are mediated by two distinct mechanisms, with centrally mediated increased descending inhibition from the rostral ventromedial medulla contributing to generalized hypoalgesia (Zhang et al., 2010; Walker et al., 2015), while the restricted ipsilateral distribution of reincision hyperalgesia is spinally mediated.

The present study provides further evidence for the role of microglia in persistent effects of early-life injury and the transition from acute to chronic pain following subsequent injury. In addition, we identify a novel preventive mechanism: pharmacological microglial inhibition at the time of neonatal injury prevented subsequent reincision hyperalgesia in a sex-dependent manner. Following preterm birth, male sex is an independent risk factor for adverse neurodevelopmental outcome (Linsell et al., 2018), but repeated procedural pain exposure has a greater impact on brain volume and connectivity in females (Schneider et al., 2018), and both somatosensory function and pain experience differ in young adult males and females born extremely preterm (Walker et al., 2018). In later life, chronic pain conditions are more prevalent in females (Mogil, 2012; Fillingim, 2017). From a clinical perspective, our data highlight the need to consider early-life experience when assessing risk for persistent pain in later life, and to compare efficacy in males and females enrolled in clinical trials of microglial inhibitors (Tong et al., 2012). In line with National Institutes of Health Federal Pain Research Strategy priorities (Price et al., 2018), our data identify an important contribution of early-life experience to pain in later life and further highlight the importance of sex as a biologic variable when evaluating mechanism and efficacy of therapeutic interventions in preclinical pain research.
